# Clinical and imaging-based prognostic factors in radioembolisation of liver metastases from colorectal cancer: a retrospective exploratory analysis

**DOI:** 10.1186/s13550-017-0292-1

**Published:** 2017-05-23

**Authors:** Kathy P. Willowson, Aimee R. Hayes, David L. H. Chan, Michael Tapner, Elizabeth J. Bernard, Richard Maher, Nick Pavlakis, Stephen J. Clarke, Dale L. Bailey

**Affiliations:** 1 Institute of Medical Physics, School of Physics, University of Sydney, Camperdown, NSW Australia; 2 Department of Nuclear Medicine, Royal North Shore Hospital, St Leonards, NSW Australia; 3 Research and Development, Sirtex Medical Limited, North Sydney, Australia; 4 Department of Radiology, Royal North Shore Hospital, St Leonards, NSW Australia; 5 Department of Medical Oncology, Royal North Shore Hospital, St Leonards, NSW Australia; 6 Sydney Medical School, University of Sydney, Camperdown, NSW Australia; 7 Faculty of Health Sciences, University of Sydney, Camperdown, NSW Australia

**Keywords:** ^90^Y, Radioembolisation, SIRT, Dose, Response, Heterogeneity

## Abstract

**Background:**

The aim of this study was to investigate the relationship between absorbed dose and response of colorectal cancer liver metastases treated with [^90^Y]-resin microspheres and to explore possible clinical and imaging derived prognostic factors.

**Methods:**

FDG PET/CT was used to measure response of individual lesions to a measured absorbed dose, derived from post-treatment ^90^Y PET imaging. Predicted dose was also derived from planning [^99m^Tc]-MAA SPECT data. Peak standardised uptake value and total lesion glycolysis (TLG) were explored as response measures, and compared to dose metrics including average dose (*D*_avg_), biologically effective dose, minimum dose to 70% of lesion volume and volume receiving at least 50 Gy. Prognostic factors examined included baseline TLG, RAS mutation status, FDG heterogeneity and dose heterogeneity. In an exploratory analysis, response and clinico-pathological variables were evaluated and compared to overall survival.

**Results:**

Sixty-three lesions were analysed from 22 patients. Poor agreement was seen between predicted and measured dose values. TLG was a superior measure of response, and all dose metrics were significant prognostic factors, with a *D*_avg_ of ~50 Gy derived as the critical threshold for a significant response (>50% reduction in TLG). No significant correlation was found between baseline TLG or RAS mutation status and response. Measured dose heterogeneity was a significant prognostic factor and when combined with *D*_avg_ had a positive predictive value for response >80%. In the exploratory analysis for prognostic factors of survival, low hepatic tumour burden and mean reduction in TLG >65% were independently associated with improved overall survival.

**Conclusions:**

Lesions receiving an average dose greater than 50 Gy are likely to have a significant response. For lesions receiving less than 50 Gy, dose heterogeneity is a significant prognostic factor. Lesions receiving an average dose less than 20 Gy are unlikely to respond. A reduction in TLG may be associated with improved overall survival.

**Electronic supplementary material:**

The online version of this article (doi:10.1186/s13550-017-0292-1) contains supplementary material, which is available to authorized users.

## Background

Radioembolisation with yttrium-90 (^90^Y)-labelled resin microspheres (SIR-Spheres, Sirtex Medical Ltd, Sydney, Australia) is a palliative treatment for metastatic colorectal cancer (mCRC) in the liver. Historically, SIR-Spheres microspheres treatment planning developed as an empirical approach, derived from a combination of patient body surface area, tumour burden and estimates of arterio-venous shunting. With recent advances in imaging hardware and software, it has become possible to image the therapeutic microspheres in vivo and quantify absorbed dose to lesions and healthy parenchyma [1, 2]. As a result, derivation of the relationship between absorbed dose and response to treatment could see radioembolisation move to a personalised approach where treatment doses are tailored to the individual for optimal outcome.

There are several published examples of lesion absorbed dose measured with ^90^Y PET being compared to treatment response [3–12]. Two apparent issues when considering the existing literature are the limited study numbers and the discordance between analysis techniques. Additionally, the literature is divided further into users of glass microspheres (TheraSpheres, BTG International Canada Inc., Ottawa, Canada) versus resin microspheres (SIR-Spheres), which will have inherent differences in the dose–response profile due to their different specific activity, quantity administered and lodgement pattern. Some studies have combined patients with different pathological diagnoses to improve statistical power however it is highly likely that the dose–response will be pathology dependent [13]. In addition, there is evidence in the literature that mutant RAS cell lines (so-called as they were initially identified in “rat sarcoma” cells) are more radio-resistant compared to wild type RAS [14, 15]; a difference that has recently been shown to affect overall survival following radioembolisation [16, 17], suggesting a more detailed approach to accurate dose–response profiling is needed.

Traditionally, response has been measured using X-ray CT to gauge anatomical change in lesion dimensions (RECIST) [18]. More recently it has been recognised that metabolic FDG PET imaging may be more suitable to measure functional response to therapy at an early time point to allow switching to a more efficacious treatment [19]. However, even within those studies that use FDG PET as a biomarker of response, there is little consistency between time to follow-up imaging and methods of lesion segmentation and the quantification indice employed. Furthermore, there is little consensus on the subject of absorbed dose measures and which metric is most meaningful. The review from Cremonesi et al. provides some insight into the large variation of factors in the existing radioembolisation dosimetry literature [20]. The use of FDG PET to characterise lesions at baseline has also been recognised as having potential. Recent literature suggests that baseline TLG may correlate with outcome [9], and in other pathologies and treatments, intra-tumoural heterogeneity of FDG has been demonstrated to be a prognostic marker and indicator of aggressiveness of disease [21–25]. To our knowledge, this has not yet been investigated for radioembolisation.

In terms of minimum radiation dose required to effect treatment, the range is reported as being as large as <50–495 Gy [20], and methodologies vary. The most extensive study to date is that from van den Hoven et al. [9], which included analysis of 133 lesions from 30 patients with unresectable mCRC. The study used change in FDG PET total lesion glycolysis (TLG) as a measure of response at 1 month post-treatment with resin microspheres and reported an effective mean absorbed dose to be 40–60 Gy. Kao et al. [6] explored the use of various dose metrics borrowed from external beam radiotherapy (EBRT) available with the generation of dose volume histograms (DVHs), such as minimum dose to 70% of the lesion volume (D_70_) and lesion volume receiving at least 100 Gy (V_100_). For hepatocellular carcinomas (HCC) treated with resin microspheres, a complete response was generally achieved for D_70_ values greater than 100 Gy based on response from CT or MRI measures with a median follow-up time of 5.4 months. Srinivas et al. [11] also reported on the value of ^90^Y dosimetry for HCC lesional assessment, finding a mean dose of 215 Gy for responders as measured on CT, based on a cohort of 98 lesions treated with glass microspheres. Most recently, Fowler et al. [10] reported an average dose of 29.8 Gy and D_70_ of 42.3 Gy as useful predictors of response as measured by volumetric MRI analysis, based on a study of 9 mCRC patients (all treated with resin microspheres) with response measured at various intervals corresponding to ‘standard of care’.

Additional estimates of effective lesion absorbed dose have been made based on dosimetry performed on the ^99m^Tc-macroaggregated albumin (MAA) planning study, done prior to treatment. However, the literature is divided as to whether or not the MAA SPECT is a suitable predictor of subsequent therapeutic microsphere lodgement [6, 26, 27]. Confounding factors may be the placement of the catheter, delivery technique, flow dynamics and differing lodgement patterns due to variation in particle shape and size, as well as vascular changes that may occur between the time of work-up and therapy.

The mechanism by which the microspheres lodge in the vasculature and the corresponding heterogeneity of radiation dose has been implicated as an additional challenge in radioembolisation dosimetry. It is recognised that this heterogeneity leads to a greater sparing capacity of normal liver and thus tolerance of higher average doses due to the non-uniformity of the dose pattern [28, 29]. Fowler et al. acknowledged the importance of considering DVHs as opposed to just average lesion dose in order to take into account, to some extent, the heterogeneity in dose delivery across the lesion [10]. This has been further confirmed by Pasciak et al. [30] who recently found a correlation between D_70_ and microsphere density, and also found that an increase in average tumour dose could compensate for poor uniformity effects.

This retrospective study investigates the dose–response relationship in the treatment of mCRC with ^90^Y resin microspheres, exploring the effectiveness of various dose and response metrics, as well as the impact of a range of prognostic factors available at baseline from both imaging and clinical data.

## Methods

### Patient selection

Thirty-six consecutive mCRC patients treated with resin microspheres between July 2011 and June 2016 were identified retrospectively. Eligibility for therapy included histologically confirmed mCRC, hepatic dominant disease, unresectable and progressive disease despite chemotherapy, preserved liver function (defined as bilirubin <34 μmol/L, albumin >30 g/L, INR < 1.1) and a pulmonary shunt fraction on MAA SPECT not exceeding 20%. Survival analysis was performed on 32 patients (one patient was lost to follow-up and three patients received repeat radioembolisation therapy at a later date) with exploratory analyses performed to determine predictors of survival. Table 1 summarises the patient characteristics.

**Table 1:** Characteristics of study cohort at baseline (*n =* 32)

Characteristic	*n* (%) unless indicated otherwise
Age in years, median (range)	63 (37–86)
Sex	
Male	23 (72%)
Female	9 (28%)
BMI, median (range)	26 (20–38)
Primary site	
Colon	23 (72%)
Rectum	9 (28%)
Definitive treatment to primary tumour	26 (81%)
Hepatic tumour burden^a^, median (range)	9 (1–46)
Hepatic tumour burden^a^ > 25%	4 (13%)
Extrahepatic metastases^b^	23 (72%)
Location of extrahepatic metastases	
Lungs alone	13
Lung and lymph nodes	2
Lymph nodes alone	5
Other	3
Prior therapies	
Lines of chemotherapy 1	9 (28%)
2	22 (69%)
Anti-VEGF/EGFR mAb	28 (88%)
Hepatic resection	11 (34%)
RFA/SBRT^c^ to liver	2 (6%)
Isolated liver oxaliplatin	1 (3%)
Radiotherapy to extrahepatic sites	6 (19%)
RAS mutation status^d^	
Wild type	14 (44%)
Mutant	10 (31%)
Unknown	8 (25%)
Treatment to both liver lobes	19 (59%)
Prescribed amount of ^90^Y for treatment in GBq, median (range)^e^	1.68 (0.42–2.08)
Time to follow-up FDG PET/CT^e^ in days, median (range)	56 (38–80)

^a^As measured by the MeVis® radiological service (MeVis Medical Solutions AG, Bremen, Germany), based on contrast enhanced CT performed at the time of baseline FDG PET/CT

^b^Detected on FDG PET/CT

^c^RFA, Radiofrequency ablation; SBRT, stereotactic body radiation therapy

^d^KRAS/NRAS where tested

^e^From sub-cohort of 22 patients with analysable lesion data only

Complete imaging sets suitable for lesional analysis were available in 22 patients. An individual complete imaging set consisted of baseline FDG PET/CT (acquired ≤ 28 days prior to radioembolisation), ^90^Y PET/CT (acquired within 24 h of radioembolisation), and follow-up FDG PET/CT (acquired ≤ 80 days post-radioembolisation). Lesions were considered analysable if they were FDG-avid and could be defined as a discrete volume on the baseline FDG PET study (as opposed to a contiguous mass of lesions), of at least 5.0 cm^3^ [31]. Up to five lesions (defined as the five with the highest standardised uptake value (SUV)) from any single patient were considered. Three patients had chemotherapy between radioembolisation and follow-up FDG PET/CT yet were include for statistical power.

### Image analysis

All imaging data were acquired on a Siemens Biograph mCT-S (64) PET/CT system (Knoxville, TN, USA) with time-of-flight (ToF) capabilities (550 ps timing resolution), 21.8 cm axial field of view and 78 cm crystal ring diameter. Standard OSEM reconstruction was used in conjunction with ToF modeling and point spread function recovery. Baseline and follow-up FDG data were acquired according to our ‘low-dose protocol’, as two 6 min frames over the liver and reconstructed with 3i21s and a 5 mm Gaussian filter. All ^90^Y PET data were acquired as two 10 min frames over the liver, and reconstructed with 1i21s with no filtering for quantitative purposes. The MAA planning SPECT/CT data were acquired on either a Siemens’ Intevo-6 or Symbia. T16 system, with low energy parallel hole collimators and underwent standard CT-based attenuation correction during reconstruction. Due to the location of the angiography suite adjacent to the nuclear medicine department, acquisition was performed immediately (in all cases within 1 h) following implantation to avoid breakdown of the ^99m^Tc-MAA in vivo.

All data analysis was performed on a DOSIsoft^®^ system (Cachan, France). Baseline FDG PET/CT, quantitative ^90^Y PET (see [32]) and follow-up FDG PET/CT studies were co-registered to the baseline contrast-enhanced CT. Where available (19 of the 22 patients), the planning [^99m^Tc]-MAA SPECT/CT data were also co-registered.

Quantitative ^90^Y PET data were used to generate treatment dose maps in units of Gy, derived through kernel-based convolution (DOSIsoft^®^), and DVHs for analysis. Predicted dose maps from the MAA SPECT were also generated, assuming an identical mapping of radioactivity distribution between the MAA and therapeutic microspheres, and that the complete prescribed amount of radioactivity would be delivered at treatment. Lesions were defined on the baseline FDG using semi-automatic detection followed by a 30% thresholding within the bounded volume. Lesion volumes were propagated on to the co-registered MAA predicted dose map and ^90^Y treatment dose map. Each lesion was located on the follow-up FDG PET, and if still detectable above background liver was re-defined, or if no longer detectable, was assigned a volume of 0 cm^3^.

The contrast-enhanced CT was used to define whole liver, before subtracting the sum of all FDG-defined metastases, to attain a volume of interest representing healthy liver. This volume was also propagated on to each of the dose maps.

Parameters of interest for each lesion were:

- Baseline FDG: SUV_peak_ (defined as the highest average of a 1mL spherical kernel centred on voxels within the lesion VOI), FDG heterogeneity (defined as the coefficient of variation (CoV) within the lesion VOI), total lesion glycolysis (TLG);
- MAA-predicted dose map: mean absorbed dose (D_avg_), minimum dose to 70% of the lesion volume (D_70_), lesion volume receiving at least 50 Gy (V_50_), biologically effective dose (BED [20]);
- ^90^Y treatment dose map: D_avg_, D_70_, V_50_, BED and dose heterogeneity within the lesion (CoV);
- Follow-up FDG: SUV_peak_ and TLG.

The D_avg_, D_70_, V_50_ and BED of healthy liver were also recorded.

**Fig. 1:**
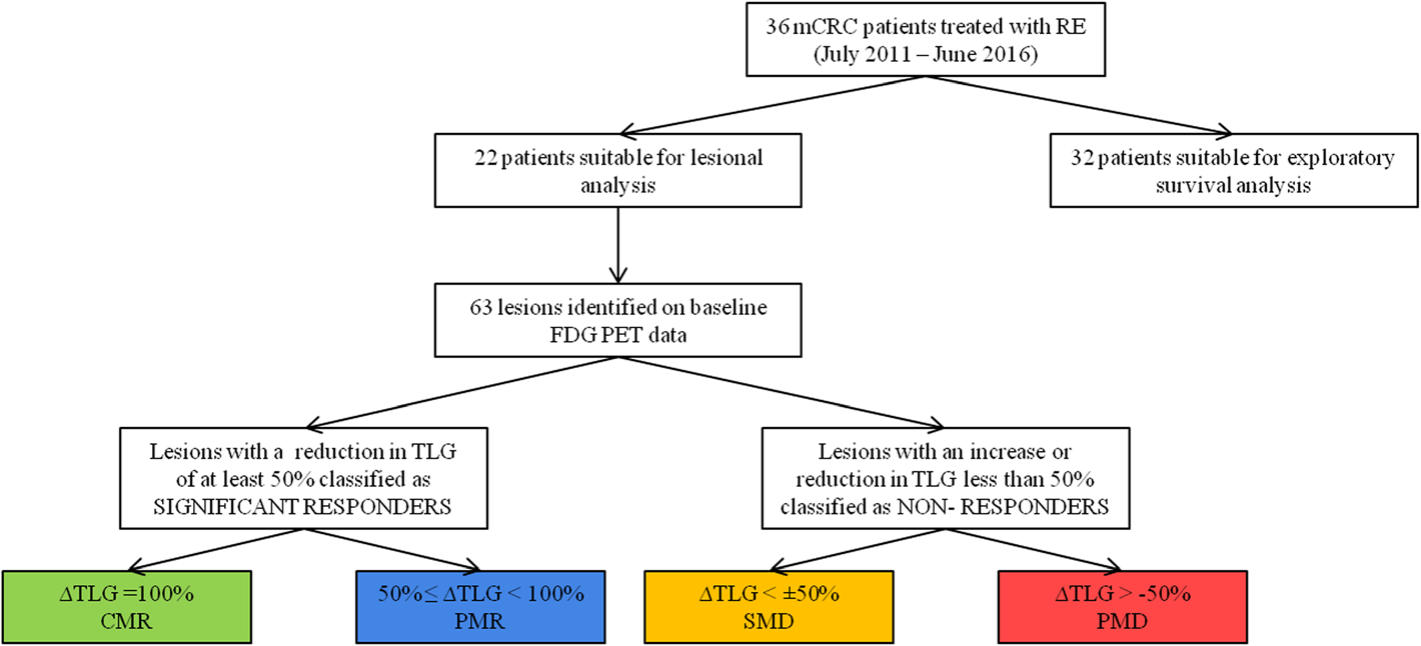
Flowchart representing the patient cohort for analysis and categorisation definitions

Treatment response was measured as both the change in SUV_peak_ and the change in TLG. Lesions were further categorised according to definitions of complete metabolic response (CMR: 100% reduction in TLG, i.e. lesions not visible above background at the time of follow-up), partial metabolic response (PMR: a reduction of TLG between 50% and 100%), stable metabolic disease (SMD: lesions that had a TLG change by less than ±50%), and progressive metabolic disease (PMD: more than a 50% increase in TLG). A significant response was classified as a reduction of at least 50% in TLG between baseline and follow-up (i.e. CMR + PMR). Figure 1represents a flow chart of cohort classification used for analysis.

Due to trends seen in the data and suggestions of both a critical threshold and effects of heterogeneity in the literature, the analysis was further divided between lesions receiving above and below an average dose of 50 Gy. For D_avg_ <50 Gy, the CoV of absorbed dose was used to predict response, based on a threshold CoV derived from the mean of responders versus non-responders.

### Statistical analysis

Statistical analyses were performed using SPSS (version 24, SPSS Inc.) and SAS (version 9.4). Significance of prognostic factors was tested using univariate binary logistic regression with a 95% confidence interval (CI) to establish those parameters which had a *p* value of less than 0.05 for predicting a significant TLG response. Those parameters with a *p* value of 0.2 or less were retained in the multivariate model. A multivariate backward stepwise likelihood ratio regression analysis was performed, excluding parameters with high collinearity (D_avg_, D_70_, V_50_ and BED), verified by consideration of both clinical factors and correlation coefficients. Four separate backward elimination procedures including each of the collinear variables in turn were performed to establish the best fitting model and identify significant predictors of response. Multivariate analysis was repeated on each sub-group of lesions that received less than 50 Gy and greater than 50 Gy (average).

Overall survival was estimated by the Kaplan-Meier method, and curves were compared using the log-rank test. Univariate and multivariate analyses were performed on both patient cohorts (entire cohort *n =* 32 and sub-cohort *n =* 22) using the variables hepatic tumour burden, presence of extrahepatic disease, age, gender, primary site of disease, resection of primary tumour, body mass index (BMI) and RAS mutation status using the Cox proportional-hazards ratio. Continuous variables were assessed as quartiles by event and optimal cut-points were explored using R version 2.15.0 (2012-03-30). On the sub-cohort of 22 patients included in the dose–response analysis, change in mean TLG after radioembolisation was also included in the univariate and multivariate analyses.

## Results

### Patients

Twenty-three men and 9 women were included in the study. Seventy-two percent had a primary colon tumour, and 28% had a primary rectal tumour. Eighty-one percent had previous definitive therapy to the primary tumour with either surgical resection or chemo-radiotherapy. Four patients had hepatic tumour burdens > 25% as measured by MeVis, and 23 patients (72%) had evidence of extrahepatic disease on baseline FDG PET/CT, predominantly involving the lungs. The majority of patients had received two lines of chemotherapy (69%) including bevacizumab or cetuximab (88%). Thirty-one percent were RAS mutation positive. Median follow-up was 9 months (range 1–62).

### Dose–response measures

**Fig. 2:**
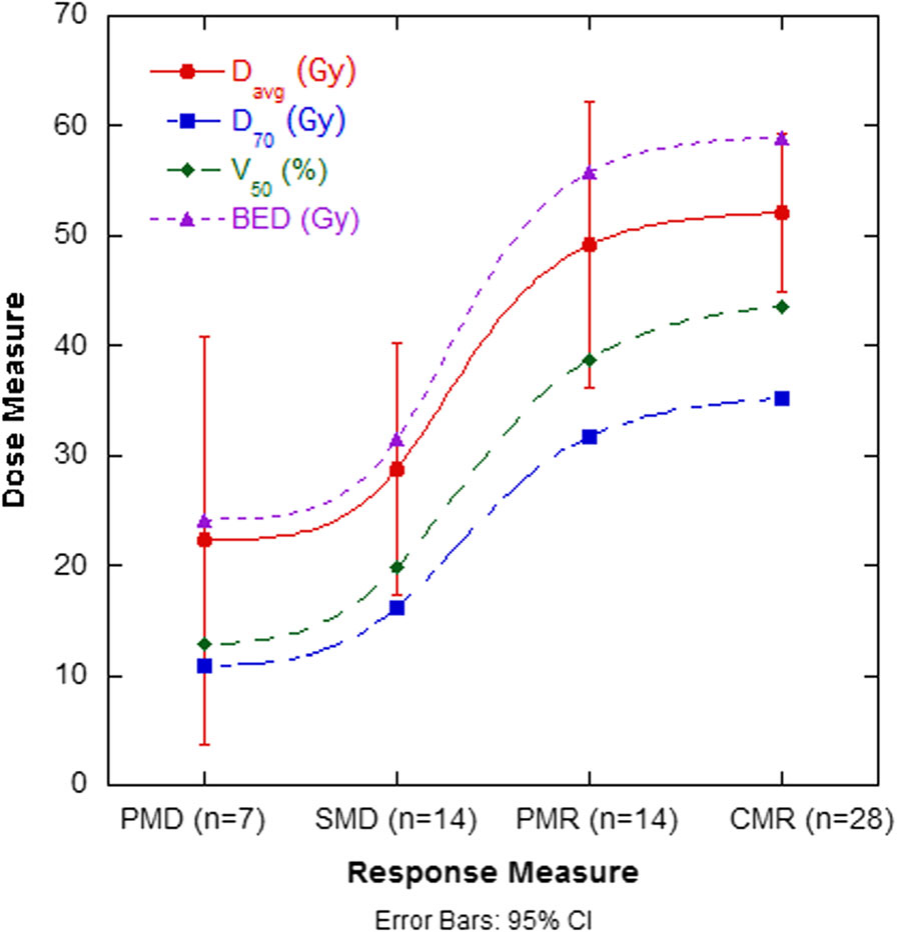
Dose curves for each category of response for D_avg_, D_70_, V_50_ and BED. *Error bars* represent the 95% CI for D_avg_

Across the 22 patients included in the lesional analysis, the average absorbed dose to healthy liver was 23.4 Gy (range: 3.9–48.5 Gy). No incidents of radioembolisation-induced liver disease (REILD) [33] were reported. Of the 63 lesions analysed, 67% demonstrated a significant response to therapy, and 89% of lesions achieved a result of SMD or better. Table 2 demonstrates the results for each category of responders. Figure 2 represents a comparison between the mean absorbed dose measured in each response category, for the different dose metrics considered. A significant difference between all investigated dose metrics of responding lesions and non-responding lesions was found (Table 3).

**Table 2:** Response rates of lesions for different ^90^Y absorbed dose metrics, where dose values are presented as Gy ± stdev

	D_avg_	D_70_	V_50_	BED
PMD (*n =* 7)	22.3 ± 20.1	10.9 ± 11.1	12.8 ± 16.6	24.2 ± 22.5
SMD (*n =* 14)	28.8 ± 19.7	16.2 ± 14.2	19.9 ± 22.7	31.5 ± 22.9
PMR (*n =* 14)	49.2 ± 22.7	31.7 ± 16.7	38.8 ± 24.5	55.8 ± 28.6
CMR (*n =* 28)	52.1 ± 18.5	35.2 ± 15.1	43.6 ± 25.8	59.0 ± 22.9
Total (*n =* 63)	42.9 ± 22.8	27.5 ± 17.4	33.9 ± 26.3	48.3 ± 27.4

**Table 3:** The mean dose (Gy ± stdev) corresponding to responding and non-responding lesions for each of the investigated metrics, including the *p* value from the two-sided *t* test of significance (set at 0.05)

	D_avg_	D_70_	V_50_	BED
Responders (*n =* 42)	51.1 ± 19.7	34.0 ± 15.5	42.0 ± 25.2	58.0 ± 24.6
Non-responders (*n =* 21)	26.6 ± 19.6	14.5 ± 13.2	17.5 ± 20.7	29.1 ± 22.5
*p* value	1.8 × 10^−5^	6.0 × 10^−6^	2.9 × 10^−4^	3.0 × 10^−5^

**Fig. 3:**
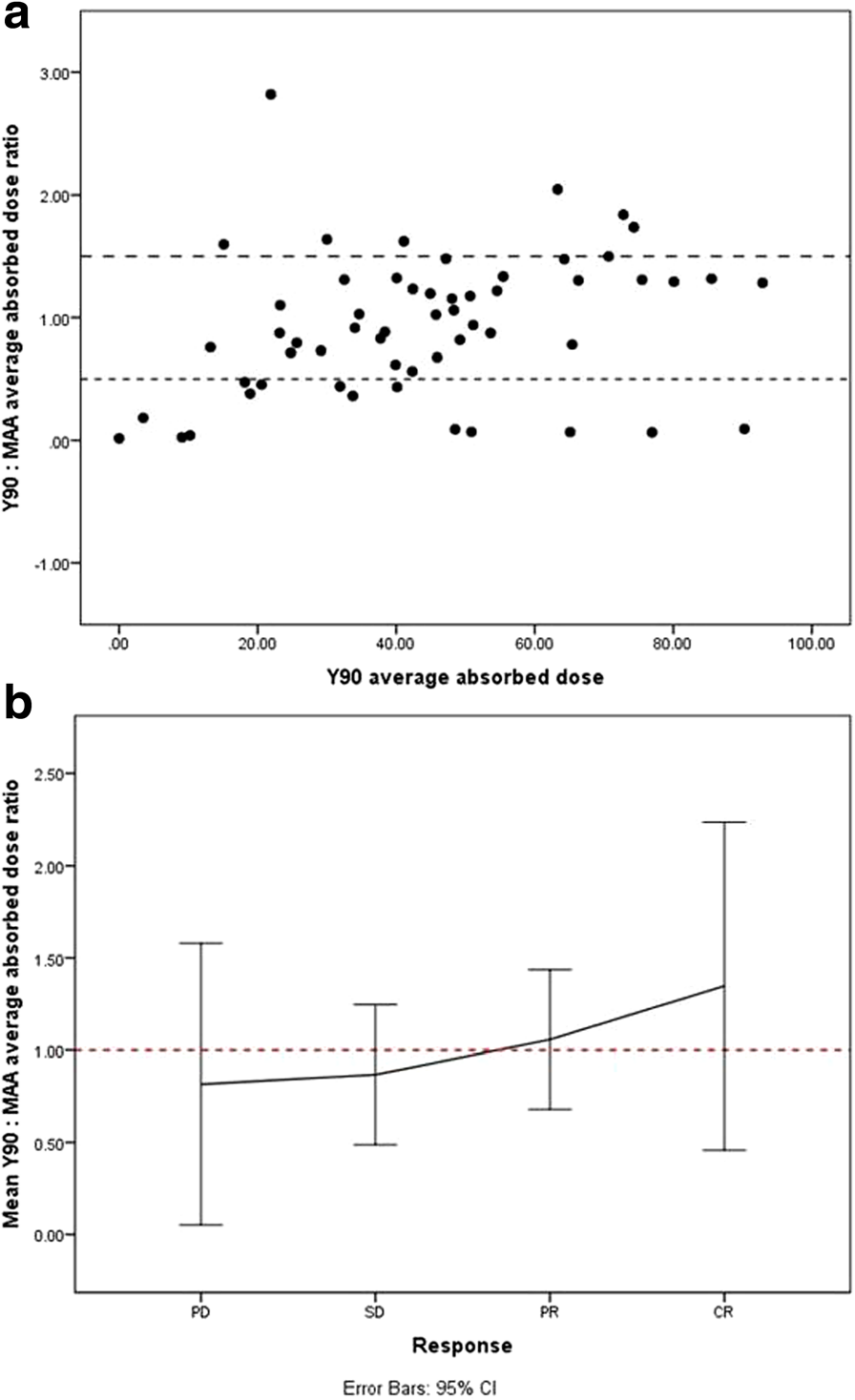
**a** The ratio of true (^90^Y) to predicted (MAA) mean absorbed dose across all lesions. *Horizontal dashed lines* represent a ±50% difference. An outlier with a ratio value of 12.3 was excluded for visual purposes. **b** The mean ratio of true (^90^Y) to predicted (MAA) lesion absorbed dose for each response category. *Dashed line* represents a ratio of 1.0

Figure 3a represents the ratio of measured to predicted D_avg_ values, through comparison of the MAA and ^90^Y data. On average, the measured D_avg_ was found to be 1.32 times higher than that which was predicted at the time of work-up (median *=* 0.93, range 0.02–12.27). Figure 3b shows the change in ratios for different response groups, demonstrating a trend towards an increased ^90^Y-to-MAA factor for improved response. Of the lesions analysed, 40% had a ^90^Y defined absorbed dose that differed by more than 50% of that which was predicted by the MAA.

**Fig. 4:**
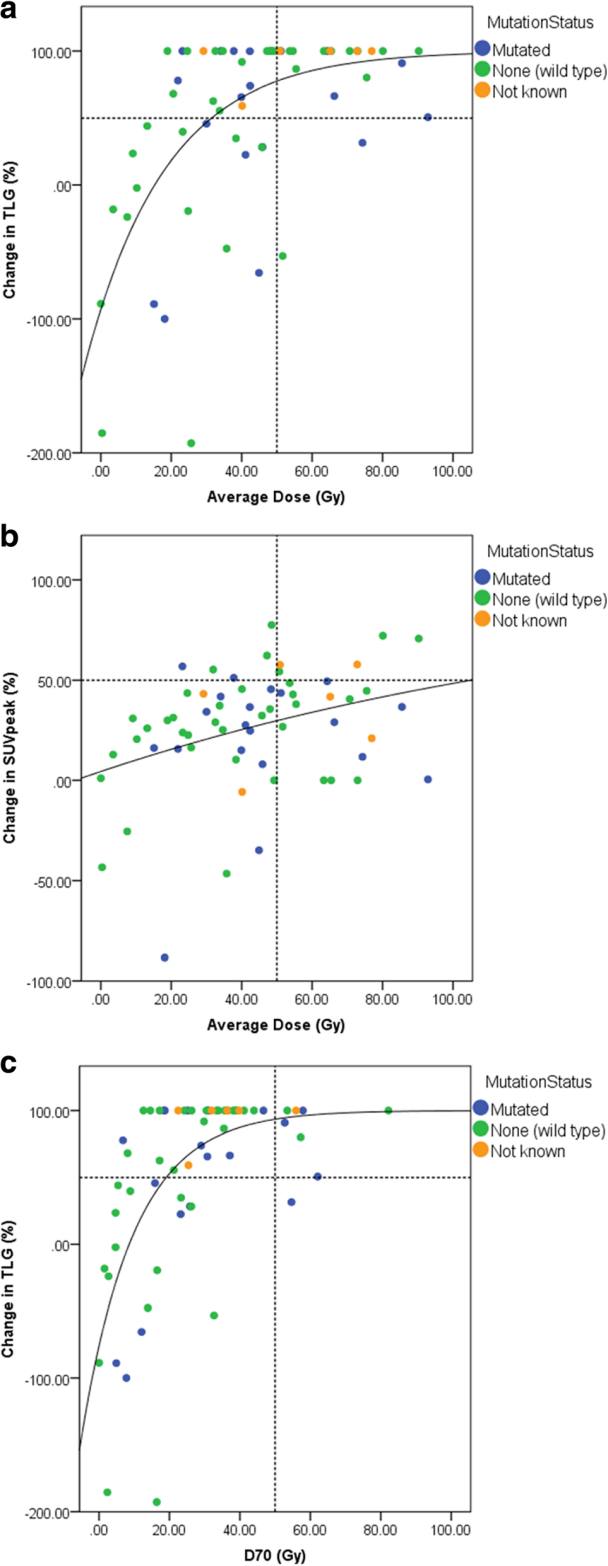
The dose–response for all lesions follows the expected relationship when measuring response with change in TLG (*R* = 0.61, when fitted with a function of the form: $y=100- a{e}^{- bx}$) (**a**); this relationship is not demonstrated when change in SUV_peak_ is used to measure response (*R* = 0.33) (**b**). Using D_70_ instead of D_avg_ as the metric slightly improves the correlation further (*R* = 0.63) (c). A 50 Gy cut-off (*dashed line*) corresponds to a significant response in all but two lesions (**a**)

Figure 4 is a comparison of SUV_peak_ and TLG as response measures (4a, b), as well as a comparison of D_avg_ and D_70_ as dose metrics (4a, c).

### Lesional response prognostic factors

**Fig. 5:**
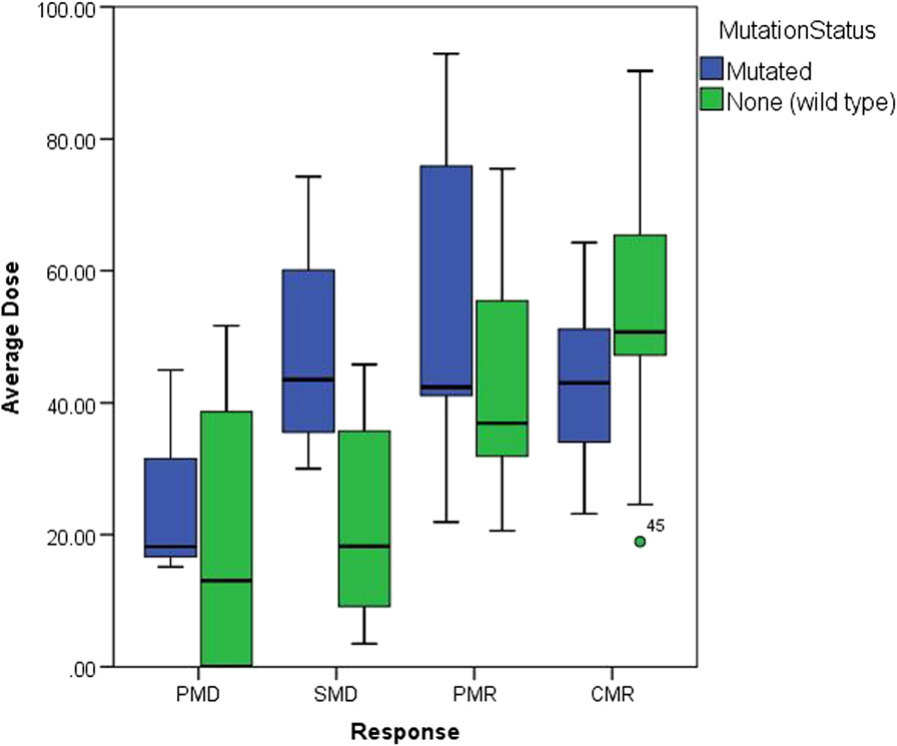
Average lesion doses corresponding to each response category, separated for lesions of wild type and mutated RAS status. The shaded regions represent the interquartile (IQ) range, with the solid line representing the median value. Mild outliers (*circle*) are points which lie greater than 1.5 times the IQ range from the upper (*3rd*) or lower (*1st*) quartile. Extreme outliers (*asterisk*) are points which lie greater than three times the IQ range from the upper (*3rd*) or lower (*1st*) quartile

Figure 5 considers the variation in D_avg_ for each response category with differing RAS status. The difference between the mean D_avg_ of responders with wild type and mutated RAS was not found to be significant. However, the average dose in non-responding lesions (SMD + PMD) was significantly higher in lesions of mutated RAS status (*p =* 0.046).

**Fig. 6:**
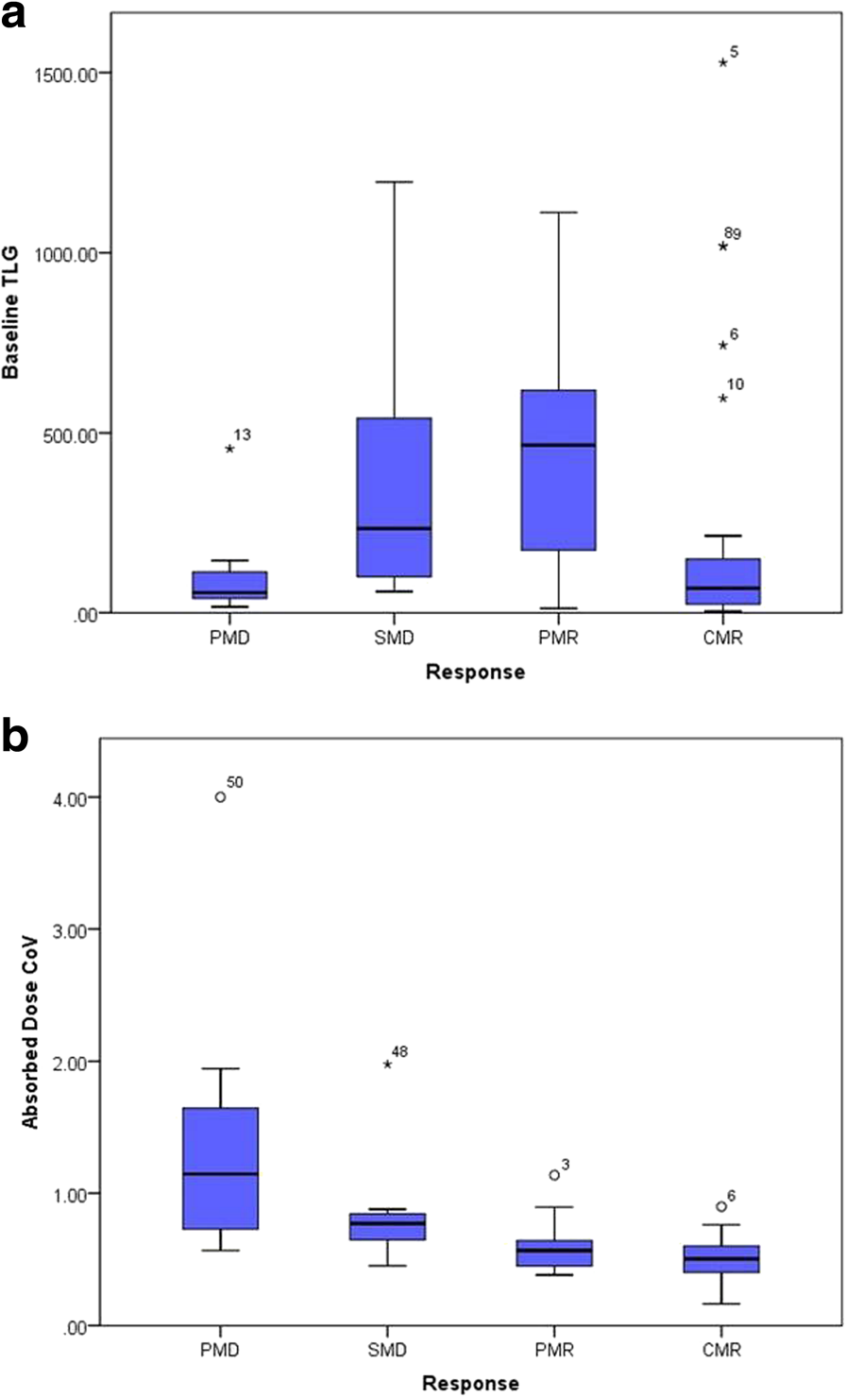
Baseline TLG (**a**) and Absorbed dose CoV (**b**) of lesions within each response category

The results of the univariate analysis can be seen in Table 4. All parameters were found to be significant when predicting response, except for baseline volume (*p =* 0.287) and baseline TLG (*p =* 0.715) (see Fig. 6a). This was also the case upon univariate analysis in the sub-group of lesions that received less than 50 Gy (average) only.

**Table 4:** Univariate (unadjusted) logistic regression and multivariate (adjusted) backward stepwise likelihood ratio regression of factors associated with significant lesional response (*)

Independent variable	*p* value			
	Unadjusted	Adjusted		
		All lesions	<50 Gy	>50 Gy
D_70_	0.0002*			
V_50_	0.001*			
D_avg_	0.0004*	0.039*	0.259	0.604
BED	0.001*			
FDG COV	0.050*	0.291	0.422	0.719
Dose COV	0.001*	0.017*	0.005*	0.868

Aside from the four dose metrics, absorbed dose CoV was the parameter found to have the strongest association with lesion response in the univariate analysis (*p =* 0.001). When absorbed dose CoV was adjusted for FDG CoV and D_avg_, the association remained statistically significant (*p =* 0.017). D_avg_ also remained a significant predictor of TLG response (*p =* 0.012) when adjusted for dose CoV and FDG CoV. Figure 6b demonstrates a clear increase in dose CoV in lesions that resulted in PMD or SMD when compared to those that demonstrate PMR or CMR. Considering the analysis of lesions receiving less than the 50 Gy threshold, dose CoV was the only significant predictor of response, whereas no variables were found to be significant prognostic factors for lesions receiving greater than 50 Gy.

Fifty-four percent of lesions in the sub-50 Gy cohort still had a significant response. We assumed all lesions receiving above 50 Gy would have a significant response, and those receiving less than 50 Gy may respond depending on absorbed dose CoV and whether or not it was above or below the mid-point between the mean of responders and non-responders (derived threshold = 0.79). A significant response was correctly predicted in 91% of >50 Gy cases (21/23 lesions), based on dose information alone. In the <50 Gy cases, the use of the above absorbed dose CoV cut-off predicted response correctly in 75% of cases (30/40 lesions). Notably, dose CoV also had the same success rate in predicting response for >50 Gy lesions (83% positive predictive value over entire lesion cohort). For the two lesions in the >50 Gy cohort that did not respond significantly, one of which was wild type and the other mutant RAS, none of the derived prognostic factors could correctly predict this outcome. This indicates that other clinical factors not explored in this work may need to be considered. The supplementary figure attached to this article provides an example of clinical case studies representing each of the categories discussed in this investigation (Additional file 1). That is, lesions receiving a high dose and demonstrating a significant response; lesions receiving a low dose and demonstrating a poor response; lesions receiving a medial dose with a low CoV and demonstrating a significant response; lesions receiving a medial dose with a high CoV and demonstrating a poor response; and lesions demonstrating a poor response despite a high dose being received.

### Exploratory survival analysis

**Fig. 7:**
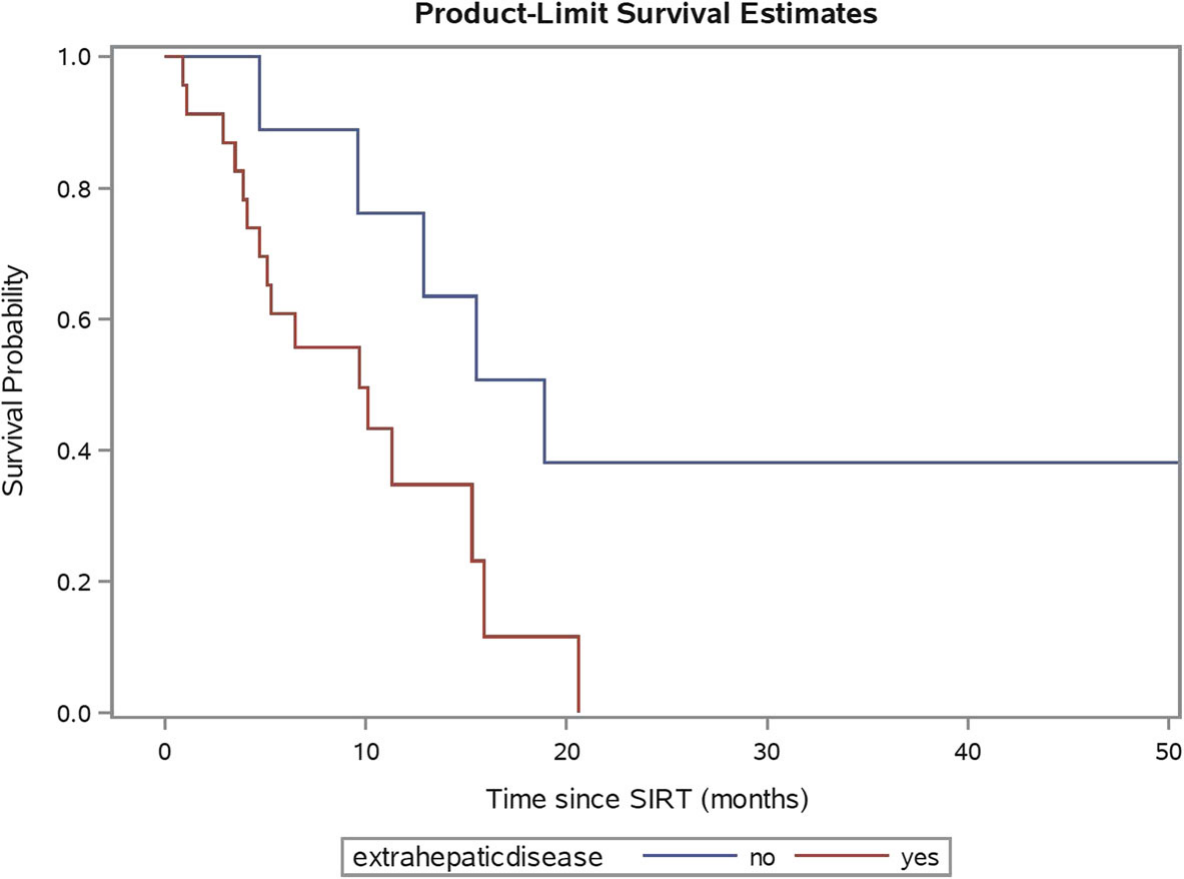
Kaplan-Meier plot of patient survival by presence or absence of extrahepatic disease on FDG PET/CT prior to radioembolisation. Patients with presence of extrahepatic disease had lower overall survival (*p =* 0.03 by log-rank test)

**Fig. 8:**
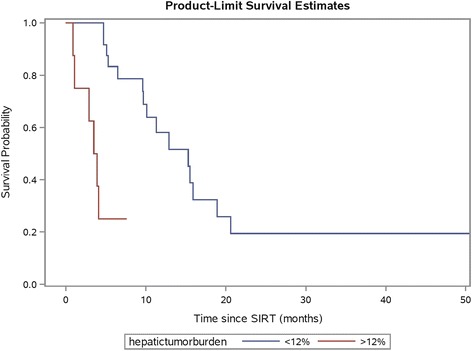
Kaplan-Meier plot of patient survival with patients dichotomised into low (≤12%) and high (>12%) hepatic tumour burden groups as measured by MeVis. Patients with hepatic tumour burden ≤12% had improved survival compared to those with higher hepatic tumour burden (*p <* 0.0001 by log-rank test)

In the entire study cohort (*n =* 32), the median overall survival after SIR-Spheres therapy was 11.3 months (95% CI 5.3–15.9 months). In univariate analyses, overall survival was significantly associated with hepatic tumour burden (*p <* 0.0001) and presence of extrahepatic disease (*p =* 0.03, Fig. 7) but not age, gender, primary site of disease, resection of primary tumour, body mass index or RAS mutation status (*p =* 0.06). After adjusting for extrahepatic disease and RAS mutation status, we found strong evidence of an independent effect of hepatic tumour burden on risk of mortality (HR 1.08, 95% CI 1.03–1.12, *p =* 0.0009). The presence of extrahepatic disease on multivariate analysis was not statistically significant (*p =* 0.06). Median overall survival in the lowest quartile of hepatic tumour burden (<6.9%) was 15.5 months compared to 3.7 months in the highest quartile (≥15.1%). In determining an optimal cutpoint, a hepatic tumour burden of 12% was found to have the highest log-rank statistic of any cut point (HR 8.24, 95% CI 2.43–27.94, *p <* 0.0001, Fig. 8).

**Fig. 9:**
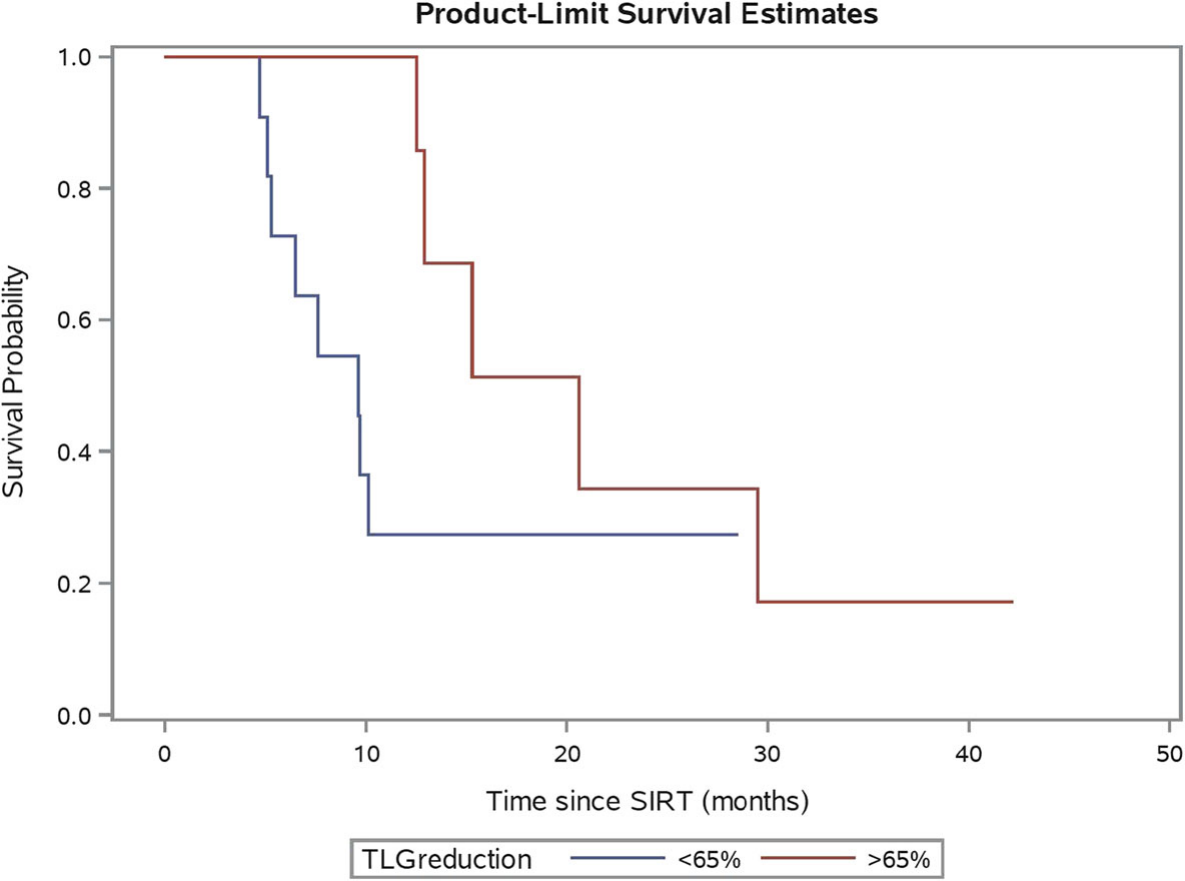
Kaplan-Meier plot of patient survival by mean TLG reduction after radioembolisation analysed in categories of >65% vs. lower. Patients with TLG reduction >65% had improved overall survival compared to those with lower TLG reduction (*p =* 0.028 by log-rank test)

In the sub-cohort of patients with data sets available for lesional dose–response analysis (*n =* 22), reduction in TLG after SIR-Spheres therapy assessed as quartiles was associated with improved overall survival on univariate analysis (*p =* 0.039). A cut point of −65% was found to have the highest log-rank test statistic (HR 3.66, 95% CI 1.07–12.53, *p =* 0.028, Fig. 9). Patients with a mean TLG reduction greater than 65% had a median overall survival of 20.6 months versus 9.6 months in those patients with a lower TLG reduction. Mean TLG reduction as categories of >65% versus lower remained independently associated with overall survival on multivariate analysis (HR 5.10, 95% CI 1.23–21.21, *p =* 0.025).

## Discussion

All dose metrics display a consistent slope and the expected sigmoidal relationship (Fig. 2). The standard deviation on dose measures for the PMD lesions is much larger than other response categories, suggesting there may be additional prognostic factors when considering which lesions will not respond. A critical average dose of ~50 Gy is consistent with other reports of effective dose for mCRC lesions responding to radioembolisation [9, 34].

Given given that our institution uses the MAA planning procedure in the capacity that is recommended by the supplier in the context of the modified BSA method, the results (Fig. 2) do suggest caution in tailoring treatments based on MAA localisation and quantification. Furthermore, the fact that the ratio of ^90^Y-to-MAA dose in lesions consistently increases with improved response (Fig. 3a) may suggest that our prescribed ^90^Y activities are underestimating the optimal dose. Similar results demonstrating inconsistency between MAA and microsphere distributions have been reported [26, 27], although similarities have been demonstrated in HCC lesions [6, 35], which is a generally more vascular pathology.

Figure 4 indicates that TLG corresponds better to the dose given compared to SUV_peak,_ which demonstrates no strong trend in the data, and thus may be a more appropriate metric to use in practice (Fig. 4a, b). SUV_peak_ was chosen due to its robustness as a quantitative metric, as opposed to SUV_max_ or SUV_mean_, which may suffer from spurious single pixel fluctuations or partial volume effects, respectively. The use of D_70_ as a dose metric may be more optimal than D_avg_ when comparing to response, as seen by a tighter clustering of points around the curve; however, the difference is subtle. The data (Fig. 4a) suggest that the D_avg_ threshold of 50 Gy is applicable when predicting lesions that will respond to radioembolisation with resin microspheres. Furthermore, the data also suggest that lesions receiving less than 20 Gy will not respond to treatment.

A larger cohort is needed to draw significant conclusions about the role that mutant RAS may play in radio-resistance of lesions to radioembolisation. The data in Fig. 4a demonstrates some outliers of mutant RAS status at high dose levels. Figure 5 also suggests that mutant RAS lesions remain at only PMD/SMD/PMR status despite much higher median doses in these categories when compared to wild type lesions, supported by the significantly higher doses seen in non-responding lesions of mutated RAS status. This may indicate that additional factors other than dose play a role when determining whether or not a mutant RAS lesion will respond. This may include the amount and timing of prior chemotherapy, including the use of anti-EGFR monoclonal antibodies, all of which may potentially radio-sensitise tumours. Both mutant RAS and wild-type lesions had the same rate of response when considered separately (~65%).

The lack of a significant relationship between baseline TLG and response was unexpected, given recent findings in the literature [9]. This may be due to the smaller cohort for analysis in this study. Additionally, an analysis of tumour-to-liver ratio (leasion SUV_peak_: healthy liver SUV_mean_) demonstrated no significant correlation with response. Of interest, however, was the significance of FDG CoV and absorbed dose CoV as prognostic factors. FDG CoV has been recognised as a potential prognostic factor in various pathologies, with one paper suggesting it is more significant than traditional measures such as SUV and TLG [25]. To our knowledge, this has not yet been explored in the realm of radioembolisation and may warrant further investigation in a larger study population. It is recognised however that CoV is a limited way of evaluating tumour heterogeneity, and other forms of analysis, such as entropy measures, may be more applicable for future work.

Absorbed dose heterogeneity is a commonly acknowledged issue associated with radioembolisation due to its vascular embolic localisation mechanism, particularly in comparison to traditional methods of irradiation employing EBRT. It has been recognised that this dose heterogeneity in healthy liver can be an advantage; however, we are unaware of any measure of lesion dose heterogeneity being linked quantitatively to response in radioembolisation. This clearly has relevance for the use of glass versus resin microspheres, due to differences in specific activity and therefore different sphere density during treatment, which has a known effect on the dose coverage [30]. The use of CoV as a metric may be advantageous, as it was recently indicated that although measures such as D_70_ can be useful in indicating dose heterogeneity, the slope of the DVH also appears to be important [10]. Furthermore, the effects of inhomogeneity may be overcome by a large average dose [30]. Dose CoV was found to be significantly higher in those lesions that did not respond, and may offer insight as to why some lesions receiving a comparably low average dose still demonstrate a CMR. Reasons for increased heterogeneity of dose across a lesion will relate to the vascular pattern of the lesion and its size, as well as the amount of administered activity. No lesion receiving less than 20 Gy (average) had a significant response, implying that the radioactivity available to the lesion was not sufficient, regardless of homogeneity of coverage.

Our data suggest a prediction of mCRC lesion response may be made immediately after radioembolisation using ^90^Y PET-derived dosimetry. If imaging indicates a lesion has received greater than 50 Gy (average) then there is a high probability that it will have a significant response to treatment. This is supported by the lack of significance of any variables when statistical analysis was done in this cohort alone. If a lesion receives less than 50 Gy (average) then the absorbed dose CoV should be considered in order to predict response, based on its high significance in statistical modeling. We found that a CoV cut-off of 0.79 had a positive predictive value of 75% in ‘low dose’ lesions. Using this approach, a combination of both average lesion dose and dose CoV had a positive predictive value of 83% for the entire lesion cohort. In addition, lesions receiving less than 20 Gy (average) are unlikely to respond regardless of CoV. Opportunity exists to explore methods to enhance lesional absorbed dose such as the concomitant use of drug therapy which may radiosensitize tumours (e.g. chemotherapy +/− anti-EGFR monoclonal antibodies or bevacizumab). Further trials may also warrant the investigation of additional SIRT, radiofrequency ablation or stereotactic body radiation therapy (SBRT) to these under-treated lesions.

In the exploratory analysis of prognostic factors for survival (*n =* 22), reduction in mean TLG was associated with prolonged overall survival supporting recent data [9] which suggests optimisation of tumour dose–response can improve patient outcomes. This warrants validation in large prospective studies. Furthermore, the survival analysis highlights the equal importance of correct patient selection for treatment. In our entire study cohort (*n =* 32), most of whom were refractory to chemotherapy, high hepatic tumour burden was associated with a poor prognosis. In addition, there was a trend towards worse overall survival in patients with presence of extrahepatic disease however this did not retain significance in the multivariate analysis. These findings are supported by the recent data from the SIRFLOX study, which evaluated the addition of SIR-Spheres microspheres to first line chemotherapy in patients with mCRC [36]. The careful selection of patients for radioembolisation based on clinical parameters will help to maximise therapeutic benefit. Optimal clinical parameters, however, need to be defined in further research.

## Conclusions

There are many prognostic factors, both clinical and image-based, that may be significant when evaluating mCRC lesional response to SIR-Spheres treatment. Absorbed dose derived from ^90^Y PET imaging post-therapy is a good predictor of response, and lesions receiving an average dose greater than 50 Gy are likely to have a significant response to treatment. For lesions receiving less than 50 Gy, the heterogeneity of dose may be an important factor in predicting outcome. An average dose less than 20 Gy indicates that lesions are unlikely to have a significant response. Baseline clinical factors (including hepatic tumour burden and presence of extrahepatic disease) and an early reduction in TLG at follow-up may be prognostic for overall survival and warrant further prospective research.

## Additional file

Additional file 1: Figure S10. Case studies representing each of the scenarios discussed in the manuscript with regards to lesional dose–response: high average dose (>50 Gy) achieving a CMR (a); intermediate average dose (20–50 Gy) with a low dose CoV achieving a PMR (b); intermediate average dose (20–50Gy) with a high dose CoV achieving SMD (c); low average dose (<20 Gy) achieving PMD (d); and the anomaly of a high average dose (<50 Gy) resulting in only SMD (e). Each row represents a transverse slice through the baseline FDG PET (left), ^90^Y PET derived dosemap (*centre*), and follow-up FDG PET (*right*). The crosshairs identify the lesion of interest. (ZIP 529 kb)

## Abbreviations

BED: Biological effective dose
CMR: Complete metabolic response
COV: Coefficient of variation
CT: Computed tomography (X-ray)
D_70_: Minimum dose to 70% of a volume
D_avg_: Average dose
DVH: Dose volume histogram
FDG: Fluorodeoxyglucose
MAA: Macroaggregated albumin
mCRC: Metastatic colorectal cancer
OSEM: Ordered subset expectation maximisation
PET: Positron emission tomography
PMD: Progressive metabolic disease
PMR: Partial metabolic response
RAS: Rat sarcoma
RECIST: Response evaluation criteria in solid tumours
REILD: Radioembolisation-induced liver disease
SMD: Stable metabolic disease
SPECT: Single photon emission computed tomography
SUV: Standardised uptake value
TLG: Total lesion glycolysis
ToF: Time of flight
V_50_: Volume receiving at least 5 Gy
