# Supplementary material for: Clinical and imaging-based prognostic factors in radioembolisation of liver metastases from colorectal cancer: a retrospective exploratory analysis
Source: EJNMMI Res. 2017 May 23;7:46. doi: 10.1186/s13550-017-0292-1 (PMC5442040; doi:10.1186/s13550-017-0292-1)
Supplement: Additional file 1: Figure S10. — Case studies representing each of the scenarios discussed in the manuscript with regards to lesional dose–response: high average dose (>50 Gy) achieving a CMR (a); intermediate average dose (20–50 Gy) with a low dose CoV achieving a PMR (b); intermediate average dose (20–50Gy) with a high dose CoV achieving SMD (c); low average dose (<20 Gy) achieving PMD (d); and the anomaly of a high average dose (<50 Gy) resulting in only SMD (e). Each row represents a transverse slice through the baseline FDG PET (left), 90Y PET derived dosemap (centre), and follow-up FDG PET (right). The crosshairs identify the lesion of interest. (ZIP 529 kb) [file 13550_2017_292_MOESM1_ESM.zip › Supplementary file (fig10).doc]

**Fig.10** Case studies representing each of the scenarios discussed in the manuscript with regards to lesional dose-response: high average dose (> 50 Gy) achieving a CMR (a); intermediate average dose (20 – 50 Gy) with a low dose CoV achieving a PMR (b); intermediate average dose (20 – 50 Gy) with a high dose CoV achieving SMD (c); low average dose (< 20 Gy) achieving PMD (d); and the anomaly of a high average dose (< 50 Gy) resulting in only SMD (e). Each row represents a transverse slice through the baseline FDG PET (left), ^90^Y PET derived dosemap (centre), and follow-up FDG PET (right). The crosshairs identify the lesion of interest.
